# A prospective evaluation of the inoculation of homogenised tissue and bone biopsies in blood culture bottles for the diagnosis of orthopaedic-device-related infections

**DOI:** 10.5194/jbji-10-317-2025

**Published:** 2025-08-21

**Authors:** Ann-Sophie Jacob, Jolien Onsea, Laura Bessems, Pauline Spoormans, Georges Vles, Willem-Jan Metsemakers, Sien Ombelet, Melissa Depypere

**Affiliations:** 1 Department of Laboratory Medicine, University Hospitals Leuven, Leuven, Belgium; 2 Department of Trauma Surgery, University Hospitals Leuven, Leuven, Belgium; 3 Department of Development and Regeneration, KU Leuven, Leuven, Belgium; 4 Department of Orthopaedics, University Hospitals Leuven, Leuven, Belgium

## Abstract

Fracture-related infections (FRIs) and periprosthetic joint infections (PJIs) occur in nearly 2 % of patients with orthopaedic devices, contributing to significant morbidity and mortality. Effective microbiological diagnosis of these infections remains challenging, with the homogenisation of bone/tissue biopsies recognised as the most reliable pre-analytical procedure.

This prospective study compared the inoculation of homogenised samples into a BacT/ALERT^^®^^ VIRTUO^^®^^ system using FA and FN PLUS blood culture bottles (BCBs) with inoculation into conventional Wilkins–Chalgren broth. Bone and tissue samples collected during surgery for suspected or confirmed FRIs or PJIs were homogenised with saline and glass beads. The resulting suspension was inoculated into BCBs (14 d incubation) or into Wilkins–Chalgren broth (10 d incubation).

Among the 59 patients included, cultures remained negative in 19 cases, whereas both methods successfully identified all pathogens in 28 patients. Although sensitivity was similar between the two methods (85 % for BCB vs. 80 % for the conventional method; p=0.77 ), the BCB method demonstrated significantly higher specificity (93 % vs. 56 %; p=0.0001 ). The BCB method yielded much faster results, with 80 % of cultures turning positive within the first 2 d of incubation (median of 24.5 h). In contrast only 16 % of cultures processed with the conventional method were positive within the same time frame (median of 69.0 h; p=0.03 ). For *Cutibacterium acnes*, a minimum incubation period of 14 d was necessary with the BCB method to ensure accurate detection.

This prospective study demonstrated an enhanced method for culturing bone and tissue biopsies from patients with FRIs or PJIs using the BacT/ALERT^^®^^ VIRTUO^^®^^ system, resulting in improved specificity and the significantly faster detection of pathogenic microorganisms.

## 1 Introduction

In 2019, an estimated 178 million fractures occurred worldwide. The burden of fracture-related infection (FRI) becomes apparent when considering that approximately 1 %–2 % of all closed fracture fixations and up to 30 % of complex open tibial fractures worldwide develop an infection (Metsemakers et al., 2024; Boxma et al., 1996; Papakostidis et al., 2011). Periprosthetic joint infections (PJIs) occur in nearly 2 % of joint replacements. This number is expected to increase because of an estimated global growth in prosthetic joint replacements (Vasoo, 2018). Orthopaedic-device-related infections (ODRIs) not only account for major morbidity and mortality, but the socioeconomic burden of these complications is also enormous (Darouiche, 2004; Moriarty et al., 2022).

Despite advancements in modern microbiological culture techniques, 5 %–34 % of PJIs remain culture-negative (Tande and Patel, 2014; Berbari et al., 2007). For FRIs, studies report culture negativity ranging from 6.1 % up to 43 % using conventional tissue culture techniques (Yano et al., 2014; Kuehl et al., 2019). Most clinical microbiology laboratory methods are based on culturing bacteria on agar plates and in enrichment broth.

These methods are labour-intensive, involve sub-culturing and require daily inspection of enrichment broth (Sanabria et al., 2019; Minassian et al., 2014; Redanz et al., 2015). This may include some subjectivity, as it is arbitrary whether to consider a broth cloudy. Semi-automated methods offer objective, easier and faster alternatives. Previous studies have demonstrated the added value of inoculating tissue into blood culture bottles (BCBs) with automated growth detection. This approach has even enabled the earlier detection of slow-growing organisms, highlighting its effectiveness in improving diagnostic timelines (Minassian et al., 2014; Sanabria et al., 2019; van den Bijllaardt et al., 2019; Tarabichi et al., 2018; Roux et al., 2011). Homogenisation has been recognised as the most effective pre-analytical procedure for biopsy samples. This can be done using a bead mill or stomacher or using glass beads. The National Health Service of England proposes the use of glass beads in their “Investigation of orthopaedic implant associated infections” protocol.

The aim of this study is to compare the effectiveness of homogenisation combined with inoculation in BCBs against homogenisation and inoculation in conventional Wilkins–Chalgren broth. Unlike previous studies, which have primarily focused on tissue samples, our research includes both bone and tissue samples from patients with FRIs, offering a more comprehensive analysis. Another important difference from PJIs is the higher prevalence of anaerobes (16 % in FRIs vs. 0.5 %–13 % in PJIs) as well as the greater frequency of polymicrobial infections and Gram-negative bacilli (Depypere et al., 2022; Aggarwal et al., 2014).

The primary objective of this work is to evaluate the sensitivity of both culture methods based on the European Bone and Joint Infection Society (EBJIS) definition of PJI and the consensus definition of FRI (McNally et al., 2021; Metsemakers et al., 2018). Secondary objectives include the assessment of the specificity of the culture methods by identifying contaminant growth, analysing the time to positivity (TTP) for both methods, determining the optimal incubation period for blood culture bottles (BCBs), and comparing the TTP between tissue samples and bone biopsies.

## 2 Material and methods

### 2.1 Study design 

Patients admitted to the University Hospitals Leuven (Belgium) from 1 November 2023 to 6 August 2024 for revision surgery for either presumed aseptic failure or with suspected or proven infection were included. Demographic and clinical data were collected from patients' electronic medical records. Patients younger than 18 years were excluded.

### 2.2 Microbiological methods

A minimum of three biopsies were individually placed in a container with 5 mL of saline and 12 glass beads (Equine and Ovin laboratories). In the laboratory, samples were processed in a class II safety cabinet using the aseptic technique.

The containers were vortexed for 15 s (40 Hz). From the homogenised suspension, 1.5 mL was inoculated into BacT/ALERT FA and FN PLUS (bioMérieux, Marcy l'Etoile, France) bottles for a 14 d culture period in a BacT/ALERT^^®^^ VIRTUO^^®^^ microbial detection system (bioMérieux, Marcy l'Etoile, France), while the remaining suspension (2 mL), along with the rest of the biopsy, was inoculated into conventional Wilkins–Chalgren broth (Oxoid) for a 10 d culture period, with sub-culturing upon cloudiness or at the end of the incubation period (terminal sub-culture). The culture process included plating the medium onto agar plates. Terminal sub-cultures were performed on chocolate (CHOC) agar under both aerobic and anaerobic conditions. BCBs were only sub-cultured if they were flagged as positive by the VIRTUO^®^ instrument, with no terminal sub-culture conducted. Once flagged positive, the BCBs were not re-incubated.

Culture results were compared to the gold standards for PJI or non-PJI, according to EBJIS definition, and for FRI or non-FRI, according to the FRI consensus definition (see Tables S1 and S2 in the Supplement respectively). Growth of low-virulence microorganisms was considered relevant (i.e. pathogenic) if identical microorganisms with similar antimicrobial susceptibility testing were isolated from at least two biopsies. Growth of a virulent microorganism was considered relevant when isolated from one or more culture media.

The following cultured microorganisms were considered virulent: *Staphylococcus aureus, Staphylococcus lugdunensis,* enterococci, β -haemolytic streptococci, *Streptococcus anginosus* group, *Streptococcus bovis* group and aerobic Gram-negative bacilli. Other cultured microorganisms (e.g. *S. epidermidis*) were considered to be low-virulence pathogens. If there was growth of one low-virulence microorganism, the decision of the multidisciplinary team involved in the patient care, considering the clinical context and cytology, counted as the final diagnosis. Growth results from both aerobic and anaerobic culture bottles from one biopsy with the same pathogen were considered one positive culture result.

### 2.3 Statistical analysis

Descriptive statistics for categorical variables are presented as counts and percentages, whereas continuous variables are summarised using medians and interquartile ranges (IQRs, $p_{25}$ – $p_{75}$ ). Differences between the FRI and PJI groups were assessed using a two-tailed t test for continuous variables and a chi-squared test for categorical variables. Differences between the methods (sensitivity and specificity) were calculated and compared using McNemar's test for paired proportions. Differences in TTP were calculated using a two-tailed Mann–Whitney U test. A p value of less than 0.05 was considered statistically significant.

## 3 Results

### 3.1 Patient characteristics

A total of 59 patients were included, comprising 42 suspected cases of FRI and 17 suspected cases of PJI (Table 1). In total, 227 samples were collected, including 132 tissue samples and 95 bone samples. Among these, 15 patients with osteosynthesis and 4 patients with prosthesis were ultimately classified as non-infectious. A detailed overview of all of the included samples can be found in Table S3.

**Table 1:** Baseline patient characteristics.

Patient characteristics			
	Osteosynthesis group	Prosthesis group	p value
	n=42 (%)	n=17 (%)	
Sex			
Male	28	12	0.77
Age (years), median ( $p_{25}$ – $p_{75}$ )	56 (44–64)	70 (57–79)	< 0.001^*^
Number of samples per patient			
Tissue samples, median ( $p_{25}$ – $p_{75}$ )	2.5 (0–5)	5 (4–7)	< 0.001^*^
Bone samples, median ( $p_{25}$ – $p_{75}$ )	3 (3–3)	0 (0–1)	0.001^*^
Anatomical localisation			
Humerus	6 (14.3)		
Clavicula	1 (2.4)		
Radius/ulna	1 (2.4)		
Femur	6 (14.3)		
Tibia	15 (35.7)		
Fibula	4 (9.5)		
Femur and tibia	1 (2.4)		
Fibula and tibia	5 (11.9)		
Olecranon	2 (4.8)		
Calcaneus	1 (2.4)		
Hip		17 (100)	
Knee		0 (0)	
Shoulder		0 (0)	
Fracture type			
Open GA grade 1	2 (4.8)		
Open GA grade 2	4 (9.5)		
Open GA grade 3A	2 (4.8)		
Open GA grade 3B	1 (2.4)		
Unknown	2 (4.8)		
Closed	31 (73.8)		
Time since trauma (days), median ( $p_{25}$ – $p_{75}$ )	240 (40–670)	730 (50–3420)	0.06
Fracture healing status			
Healed	21 (50.0)		
Unhealed/non-union	21 (50.0)		
Clinical presentation			
Fistula	6 (14.3)	0 (0)	0.37
Sinus	0 (0)	0 (0)	
Wound breakdown	9 (21.4)	1 (5.9)	0.15
Purulent drainage/pus	7 (16.7)	0 (0)	0.27
Clinical signs (rubor, tumour, dolour, calour, fever)	34 (81.0)	15 (88.2)	0.5
New-onset joint effusion	2 (4.8)	2 (11.8)	0.33
Wound drainage	13 (31.0)		
Identified pathogens by culture confirmation	26 (61.9)	12 (70.6)	0.53
Identified pathogens by culture suggestion (infection likely)	1 (2.4)	1 (5.9)	0.5
Evaluated as non-infectious	15 (35.7)	4 (23.5)	0.36
Antibiotic treatment < 2 weeks before sampling	5 (11.9)	2 (11.8)	0.99
Monomicrobial culture	21 (50.0)	13 (76.5)	0.06
Polymicrobial culture	6 (14.3)	0 (0)	0.37

^*^ Statistically significant at p < 0.05. Blank cells indicate items that are not applicable.

In patients with prosthesis, almost exclusively tissue (mean of 5) samples were taken, whereas in the osteosynthesis group, an equal number of tissue (mean of 2.5) and bone (mean of 3) samples were collected. In patients with suspected FRI, the tibia was the most frequently affected anatomical site (35.7 %), whereas the hip was the sole anatomical site affected in patients with suspected PJI. The time from trauma to infection onset was notably shorter in the osteosynthesis group (240 d) compared to the prosthesis group (730 d; p=0.06 ; Table 1).

### 3.2 Detection of microorganisms

**Figure 1:**
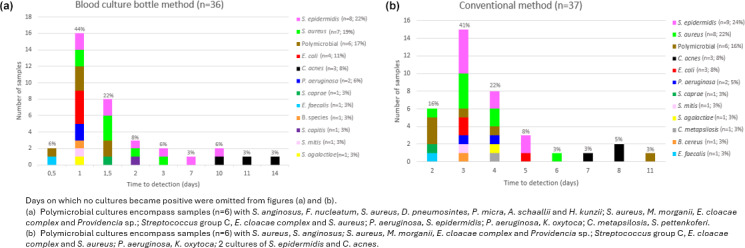
Microorganisms and the time to detection using the blood culture bottle method **(a)** and the conventional method **(b)**.

Of the 59 patients included, 19 (32 %) cultures remained negative. In 28 patients (47 %), both methods successfully detected all causative pathogens, with *S. epidermidis* and *S. aureus* being the predominant pathogens (Fig. 1). The conventional method failed to recover pathogens in seven cases (12 %). In comparison, the BCB method missed pathogens in five cases (8 %; Table 2). However, the BCB method demonstrated slightly higher sensitivity (85 % vs. 80 %; p=0.77 ). Table S4 gives an overview of all positive samples with at least one method). When considering only FRIs, the conventional method showed a somewhat higher sensitivity (96 % vs. 89 %), although this difference was not statistically significant ( p=0.6171 ).

**Table 2:** Comparison of pathogen detection using the blood culture bottle (BCB) and the conventional method.

Pathogen identified	BCB positive	BCB negative	Total
Conventional method positive	28	5	33
Conventional method negative	7	19	26
Total	35	24	59

One FRI case in which the conventional method failed to identify all pathogens involved a polymicrobial culture containing multiple anaerobic bacteria. Two additional FRI cases with discrepancies also involved polymicrobial infections: in the first case, the conventional method failed to detect *P. aeruginosa* alongside *S. epidermidis*; in the second case, there was only growth of *C. metapsilosis* but no growth of *S. epidermidis* using the conventional method. In a separate PJI case, the BCB method detected *S. capitis*, which was missed by the conventional method. Another PJI patient had *E. coli* identified in all samples using the BCB method, whereas no growth was observed with the conventional method. Additionally, in one FRI case, *S. epidermidis* was correctly identified as the causative pathogen, but the conventional method detected it in only a single sample, which would have been considered negative without confirmation from the two positive BCB samples. The patient's symptoms were compatible with a low-grade infection (e.g. pain, non-union and an elevated C-reactive protein value of 18 mg L^−1^).

For microorganisms detected with the conventional method but missed by the BCB method, one patient tested positive for *S. aureus* using the conventional approach, although only one of six collected samples was positive. Another discrepancy involved a patient for which *S. epidermidis* was missed by the BCB method.

In the specific context of the slow-growing *C. acnes* pathogen, the BCB method identified it in one case that the conventional method missed. However, the conventional method detected *C. acnes* in three cases where the BCB method failed.

To assess specificity, the frequency of at least one contaminant per patient was evaluated. No contamination was observed in 28 patients, while contamination was detected using both methods in seven cases. The BCB method showed contamination in two cultures (3 %) that were not contaminated using the conventional method. However, the conventional method yielded contamination in 22 cases (37 %), while no contaminants were detected with the BCB method (Table 3). It appears that the specificity of the new method is clearly superior to that of the conventional method ( p<0.001 ). Focusing solely on patients diagnosed with FRIs, the BCB method again demonstrated superiority, with a specificity of 96 % compared to 69 % for the conventional method ( p=0.0033 ).

**Table 3:** Contamination rates for the blood culture bottle (BCB) method vs. the conventional method.

Contamination rate	BCB – growth	BCB – no growth	Total
	of contaminant	of contaminant	
Conventional method – growth of contaminant	7	22	29
Conventional method – no growth of contaminant	2	28	30
Total	9	50	59

### 3.3 Time to positivity

For the analysis of the TTP, contaminants were excluded. Using the BCB method, 80 % (29 of 36) of the cultures became positive within the first 2 d of incubation (median of 24.5 h, $p_{25}$ – $p_{75}$ of 22–42). In contrast, only 16 % (6 of 37) of cultures processed with conventional method were positive within the same period (median of 69.0 h, $p_{25}$ – $p_{75}$ of 65.0–94.5). It took 4 d for 80 % of the conventional method cultures to show positivity, constituting a statistically significant difference ( p=0.03 ; Fig. 1).

Further analysis of bacterial detection times with the BCB method revealed that aerobic bacteria became positive within up to 10 d of incubation, while anaerobic, slow-growing bacteria required up to 14 d. For the conventional method, most anaerobic, slow-growing pathogens were detected between days 7 and 8 of incubation (Fig. 1).

In the subgroup of patients with FRIs, comparable findings were noted. The BCB method had a median TTP of 25 h ( $p_{25}$ – $p_{75}$ of 22.4–34.5), whereas the conventional method showed a significantly longer median TTP of 67.5 h ( $p_{25}$ – $p_{75}$ of 57.0–94.0), although this difference narrowly failed to reach statistical significance ( p=0.08 ). Within the first 2 d, 79 % of the cultures became positive with the BCB method, while the conventional method showed a markedly poorer performance, with only 15 % positivity within the same time frame.

A sub-analysis on both patients with FRIs and PJIs was conducted to compare TTP for tissue and bone samples using both methods. The BCB method yielded 168 positive samples (61 bone and 107 tissue), while the conventional method yielded 126 positive samples (55 bone and 71 tissue). As the conventional method has already been shown to take significantly longer to achieve positivity, comparisons between sample types were made only within each method. For the new method, bone samples had a median TTP of 32 h, while tissue samples had a median TTP of 27 h. Similarly, with the conventional method, only a small difference was observed between the two sample types. Bone samples tested positive after a median of 71.5 h, whereas tissue samples tested positive after a median of 70 h. Both differences were not statistically significant (Table 4).

**Table 4:** Comparison of bone and tissue samples analysed with both the blood culture bottle (BCB) method and the conventional method (CM) and the time to positivity (TTP).

	BCB		CM	
	Bone	Tissue	Bone	Tissue
No. of samples (without contaminants)	61	107	55	71
TTP, median (hours)	32.0	27.0	71.5	70
TTP, IQR1 (hours)	22.0	22.3	51.8	65
TTP, IQR3 (hours)	48.0	51.0	96	138
p value	0.63		0.73	

## 4 Discussion

Recent advances in microbiological diagnostics, including sonication and homogenisation techniques, have improved bacterial yield. While the IDSA (Infectious Diseases Society of America) guidelines of 2013 called for further research on bead mill processing and homogenisation, more recent studies have confirmed their added value (Osmon et al., 2013; Redanz et al., 2015; Suren et al., 2017). Another promising approach is direct inoculation into BCBs, which we evaluated for homogenised bone and tissue biopsies from FRI and PJI patients. Despite evidence supporting BCB use, previous studies excluded bone samples from FRI patients, limiting direct comparisons (Roux et al., 2011; Fang et al., 2021; Sanabria et al., 2019; Minassian et al., 2014).

Methodological variations further complicate assessments; for instance, one study used only paediatric bottles, restricting anaerobic growth, while another added horse blood, per manufacturer guidelines, to enhance fastidious organism recovery. Given the rarity of these pathogens in FRIs/PJIs and the contamination risk, we opted against this (Birdsall et al., 2021; Sanabria et al., 2019; Minassian et al., 2014; Hughes et al., 2011; Dudareva et al., 2018; van den Bijllaardt et al., 2019; Peel et al., 2016). Choosing the right anaerobic bottles is also crucial.

BacT/ALERT^^®^^ SN bottles (bioMérieux, Marcy l'Etoile, France) support anaerobic growth but lack antibiotic-absorbing resins, unlike FN PLUS bottles (bioMérieux, Marcy l'Etoile, France) that improve recovery of *Bacteroides, Clostridium* and *Prevotella* species and benefit patients pre-treated with antibiotics (Lee et al., 2013).

While one study found higher *C. acnes* recovery with SN bottles, we use the VIRTUO^®^ (bioMérieux, Marcy l'Etoile, France) incubation system and focus on FRIs, where anaerobes (excluding *C. acnes*) are more prevalent than in PJIs (Jeverica et al., 2020; Kuehl et al., 2019; Tande and Patel, 2014; Trampuz and Zimmerli, 2006; Trampuz et al., 2005). Thus, FN PLUS bottles were chosen for our study.

### 4.1 Microbiological detection 

The primary objective of this study was to compare the sensitivity of the blood culture bottle method and the conventional culture method. No significant difference was observed between the two methods, aligning with previous research that reported comparable or superior specificity for the BCB system (Bemer et al., 2016; Sanabria et al., 2019; Hughes et al., 2011; Peel et al., 2016; Birdsall et al., 2021; Velay et al., 2010). As patients with PJIs cannot be directly compared to those with FRIs due to the higher prevalence of polymicrobial cultures and anaerobic mixed flora in FRIs, this study specifically focused on the subpopulation of patients with FRIs. Within this subgroup of FRI patients, similar results were observed. The lack of superiority in this study may be attributed to the use of enrichment media and terminal sub-culturing for all samples in the conventional method.

Pathogenic microorganisms were missed in five patients with the BCB method and seven patients with the conventional method, consistent with findings by Peel et al. (2016). In one case where the conventional method failed to detect *E. coli*, the patient had received preoperative antibiotics, highlighting the BCB system's advantage of using resin-coated bottles to neutralise antibiotics. Additionally, polymicrobial cultures were identified in six patients, with the BCB method detecting a greater number of microorganisms, particularly anaerobic pathogens, in one case. Further research is needed to clarify whether the FN PLUS bottle outperforms the SN bottle with respect to detecting anaerobic pathogens other than *C. acnes*.

*C. acnes* is a slow-growing organism associated with PJI of the shoulder and the hip (Shah et al., 2015; Hedlundh et al., 2021). Due to its minimal CO_2_ production, it often produces a delayed positive signal in blood culture systems, for which a longer incubation time could be necessary (Jeverica et al., 2020; Achermann et al., 2014). When looking at this pathogen, it was missed once with the conventional method, whereas it was missed three times with the BCB method. This could potentially be attributed to the type of BCB used, although the microorganism was successfully detected in three patients.

As a secondary end point, the study evaluated the specificity of both methods. The BCB method demonstrated a significantly lower contamination rate compared to the conventional method. This observation was confirmed in the subgroup of patients with FRIs, where the BCB method also demonstrated markedly higher specificity. This advantage is likely due to the reduced sample handling in the closed BCB system, which reduces the risk of contaminating the sample during the laboratory work-up. A lower contamination rate simplifies culture interpretation and minimises the need for multidisciplinary discussions about infection status.

### 4.2 Time to positivity

The BCB method outperformed the conventional method in terms of the TTP, leveraging the BacT/ALERT^^®^^ VIRTUO^^®^^ Microbial Detection System (bioMérieux, Marcy l'Etoile, France) which is known to allow faster detection of microbial growth (She et al., 2018). In this study, the TTP was defined as the time from laboratory reception until pathogen identification was available to the treating physician, a critical metric for guiding antibiotic therapy.

While the TTP for the BCB method was significantly shorter than for the conventional method, our observed TTP values were slightly longer than those reported in some studies, which measured time to signal detection rather than pathogen identification. For the BCB method, over 70 % of bacteria were detected within the first 18 h of incubation in other studies, whereas our findings demonstrated significant improvements compared to the conventional method despite a longer TTP (Sanabria et al., 2019; Bemer et al., 2016). The BCB method's faster TTP clearly offers an advantage for timely antibiotic management. To date, no studies have reported the TTP in patients with FRIs. Consistent with earlier findings for PJIs, our results indicate that the TTP is considerably reduced when bone and tissue biopsies of patient with FRIs are inoculated into BCBs. Although this difference did not reach statistical significance, a clear trend toward a shorter TTP with the BCB method was observed. This lack of significance is likely attributable to the limited sample size in the FRI patient group, which was constrained by the mandatory exclusion of both negative and contaminated samples for this sub-analysis.

The appropriate incubation period for recovery of anaerobes remains a topic of debate. While some studies suggest that shorter durations may suffice (Rieber et al., 2018), the detection of *C. acnes* often requires longer incubation (8.7 d) (Sanabria et al., 2019). Our findings showed that *C. acnes* was detected after 11–14 d of incubation with the BCB method, supporting a minimum 14 d incubation period. This recommendation aligns with prior studies using conventional methods (Butler-Wu et al., 2011; Schafer et al., 2008). However, extended incubation periods should be critically assessed, as delayed positivity could be associated with contamination. In our patient cohort, one *C. acnes* infection was missed by the BCB method but was successfully identified through sonication culture, a technique routinely performed in our laboratory. Peel et al. (2016) demonstrated that, while longer incubation increases contamination risk, a 14 d period remains a reasonable balance for detecting slow-growing pathogens.

### 4.3 Limitations

This study has some limitations. First, it was conducted on a small scale and within a single centre. Not all bacteria that are challenging to culture were detected (e.g. *H. influenzae* and *S. pneumoniae*), limiting our ability to draw conclusions regarding these organisms. Additionally, only a small number of anaerobic bacteria were detected, providing limited evidence in this area. Nevertheless, the BCB method demonstrated the ability to detect more anaerobic bacteria in polymicrobial cultures.

A second limitation is that the available 5 mL sample had to be divided between the two methods, with 1.5 mL allocated to each BCB and approximately 2 mL to the conventional method. In routine practice, a higher volume can be inoculated into the BCBs, which would likely enhance both the sensitivity and the TTP of this method.

A third limitation is that the incubation time for the BCB method was longer than that of the conventional method. However, terminal sub-culturing was performed for the conventional method.

## 5 Conclusions

In conclusion, our prospective study demonstrated an enhanced method for culturing bone and tissue biopsies from patients with FRIs or PJIs using an automated BCB system, resulting in improved specificity and significantly faster detection of pathogens. This approach can facilitate earlier optimal patient treatment. To ensure the detection of all anaerobic bacteria, an incubation period of 14 d is required when using FN PLUS bottles.

## Supplement

10.5194/jbji-10-317-2025-supplement The supplement related to this article is available online at https://doi.org/10.5194/jbji-10-317-2025-supplement.

## Data Availability

The underlying research data are not publicly accessible due to the sensitive nature of the patient information extracted from hospital systems and privacy regulations. All relevant data concerning positive cultures are fully listed and described in the Supplement of this article (see Tables S3 and S4). Therefore, providing public access to the raw data is not necessary.

## Code availability

All of the data analyses and processing were performed using commercially available and widely used software packages (Microsoft Excel).

## Appendix

The supplement related to this article is available online at https://doi.org/10.5194/jbji-10-317-2025-supplement.

## References

[bib1.bib1] Achermann Y, Goldstein EJ, Coenye T, Shirtliff ME (2014). Propionibacterium acnes: from commensal to opportunistic biofilm-associated implant pathogen. Clin Microbiol Rev.

[bib1.bib2] Aggarwal VK, Bakhshi H, Ecker NU, Parvizi J, Gehrke T, Kendoff D (2014). Organism profile in periprosthetic joint infection: pathogens differ at two arthroplasty infection referral centers in Europe and in the United States. J Knee Surg.

[bib1.bib3] Bemer P, Leger J, Tande D, Plouzeau C, Valentin AS, Jolivet-Gougeon A, Lemarie C, Kempf M, Hery-Arnaud G, Bret L, Juvin ME, Giraudeau B, Corvec S, Burucoa C, the Centre de Reference des Infections Osteo-articulaires du Grand Ouest Study (2016). How Many Samples and How Many Culture Media To Diagnose a Prosthetic Joint Infection: a Clinical and Microbiological Prospective Multicenter Study. J Clin Microbiol.

[bib1.bib4] Berbari EF, Marculescu C, Sia I, Lahr BD, Hanssen AD, Steckelberg JM, Gullerud R, Osmon DR (2007). Culture-negative prosthetic joint infection. Clin Infect Dis.

[bib1.bib5] Birdsall J, Tambosis E, Siarakas S (2021). Isolation of clinically significant microorganisms from prosthetic joint tissue using BacT/ALERT paediatric blood culture bottles compared with solid culture media and enrichment broth. Pathology.

[bib1.bib6] Boxma H, Broekhuizen T, Patka P, Oosting H (1996). Randomised controlled trial of single-dose antibiotic prophylaxis in surgical treatment of closed fractures: the Dutch Trauma Trial. Lancet.

[bib1.bib7] Butler-Wu SM, Burns EM, Pottinger PS, Magaret AS, Rakeman JL, Matsen III FA, Cookson BT (2011). Optimization of periprosthetic culture for diagnosis of Propionibacterium acnes prosthetic joint infection. J Clin Microbiol.

[bib1.bib8] Darouiche RO (2004). Treatment of infections associated with surgical implants. N Engl J Med.

[bib1.bib9] Depypere M, Sliepen J, Onsea J, Debaveye Y, Govaert GAM, FFA IJ, Zimmerli W, Metsemakers WJ (2022). The Microbiological Etiology of Fracture-Related Infection. Front Cell Infect Microbiol.

[bib1.bib10] Dudareva M, Barrett L, Figtree M, Scarborough M, Watanabe M, Newnham R, Wallis R, Oakley S, Kendrick B, Stubbs D, McNally MA, Bejon P, Atkins BA, Taylor A, Brent AJ (2018). Sonication versus Tissue Sampling for Diagnosis of Prosthetic Joint and Other Orthopedic Device-Related Infections. J Clin Microbiol.

[bib1.bib11] Fang X, Zhang L, Cai Y, Huang Z, Li W, Zhang C, Yang B, Lin J, Wahl P, Zhang W (2021). Effects of different tissue specimen pretreatment methods on microbial culture results in the diagnosis of periprosthetic joint infection. Bone Joint Res.

[bib1.bib12] Hedlundh U, Zacharatos M, Magnusson J, Gottlander M, Karlsson J (2021). Periprosthetic hip infections in a Swedish regional hospital between 2012 and 2018: is there a relationship between Cutibacterium acnes infections and uncemented prostheses?. J Bone Joint Infect.

[bib1.bib13] Hughes HC, Newnham R, Athanasou N, Atkins BL, Bejon P, Bowler IC (2011). Microbiological diagnosis of prosthetic joint infections: a prospective evaluation of four bacterial culture media in the routine laboratory. Clin Microbiol Infect.

[bib1.bib14] Jeverica S, El Sayed F, Camernik P, Kocjancic B, Sluga B, Rottman M, Papst L (2020). Growth detection of Cutibacterium acnes from orthopaedic implant-associated infections in anaerobic bottles from BACTEC and BacT/ALERT blood culture systems and comparison with conventional culture media. Anaerobe.

[bib1.bib15] Kuehl R, Tschudin-Sutter S, Morgenstern M, Dangel M, Egli A, Nowakowski A, Suhm N, Theilacker C, Widmer AF (2019). Time-dependent differences in management and microbiology of orthopaedic internal fixation-associated infections: an observational prospective study with 229 patients. Clin Microbiol Infect.

[bib1.bib16] Lee DH, Kim SC, Bae IG, Koh EH, Kim S (2013). Clinical evaluation of BacT/Alert FA plus and FN plus bottles compared with standard bottles. J Clin Microbiol.

[bib1.bib17] McNally M, Sousa R, Wouthuyzen-Bakker M, Chen AF, Soriano A, Vogely HC, Clauss M, Higuera CA, Trebse R (2021). The EBJIS definition of periprosthetic joint infection. Bone Joint J.

[bib1.bib18] Metsemakers WJ, Morgenstern M, McNally MA, Moriarty TF, McFadyen I, Scarborough M, Athanasou NA, Ochsner PE, Kuehl R, Raschke M, Borens O, Xie Z, Velkes S, Hungerer S, Kates SL, Zalavras C, Giannoudis PV, Richards RG, Verhofstad MHJ (2018). Fracture-related infection: A consensus on definition from an international expert group. Injury.

[bib1.bib19] Metsemakers WJ, Moriarty TF, Morgenstern M, Marais L, Onsea J, O'Toole RV, Depypere M, Obremskey WT, Verhofstad MHJ, McNally M, Morshed S, Wouthuyzen-Bakker M, Zalavras C (2024). The global burden of fracture-related infection: can we do better?. Lancet Infect Dis.

[bib1.bib20] Minassian AM, Newnham R, Kalimeris E, Bejon P, Atkins BL, Bowler IC (2014). Use of an automated blood culture system (BD BACTEC) for diagnosis of prosthetic joint infections: easy and fast. BMC Infect Dis.

[bib1.bib21] Moriarty TF, Metsemakers WJ, Morgenstern M, Hofstee MI, Vallejo Diaz A, Cassat JE, Wildemann B, Depypere M, Schwarz EM, Richards RG (2022). Fracture-related infection. Nat Rev Dis Primers.

[bib1.bib22] Osmon DR, Berbari EF, Berendt AR, Lew D, Zimmerli W, Steckelberg JM, Rao N, Hanssen A, Wilson WR, Infectious Diseases Society of A (2013). Diagnosis and management of prosthetic joint infection: clinical practice guidelines by the Infectious Diseases Society of America. Clin Infect Dis.

[bib1.bib23] Papakostidis C, Kanakaris NK, Pretel J, Faour O, Morell DJ, Giannoudis PV (2011). Prevalence of complications of open tibial shaft fractures stratified as per the Gustilo-Anderson classification. Injury.

[bib1.bib24] Peel TN, Dylla BL, Hughes JG, Lynch DT, Greenwood-Quaintance KE, Cheng AC, Mandrekar JN, Patel R (2016). Improved Diagnosis of Prosthetic Joint Infection by Culturing Periprosthetic Tissue Specimens in Blood Culture Bottles. mBio.

[bib1.bib25] Redanz S, Podbielski A, Warnke P (2015). Improved microbiological diagnostic due to utilization of a high-throughput homogenizer for routine tissue processing. Diagn Microbiol Infect Dis.

[bib1.bib26] Rieber H, Frontzek A, Jerosch J, Alefeld M, Strohecker T, Ulatowski M, Morawietz T, Hinsenkamp S, Bell A, Kucukkoylu D, Frommelt L (2018). Periprosthetic joint infection caused by anaerobes. Retrospective analysis reveals no need for prolonged cultivation time if sensitive supplemented growth media are used. Anaerobe.

[bib1.bib27] Roux AL, Sivadon-Tardy V, Bauer T, Lortat-Jacob A, Herrmann JL, Gaillard JL, Rottman M (2011). Diagnosis of prosthetic joint infection by beadmill processing of a periprosthetic specimen. Clin Microbiol Infect.

[bib1.bib28] Sanabria A, Rokeberg MEO, Johannessen M, Sollid JE, Simonsen GS, Hanssen AM (2019). Culturing periprosthetic tissue in BacT/Alert(R) Virtuo blood culture system leads to improved and faster detection of prosthetic joint infections. BMC Infect Dis.

[bib1.bib29] Schafer P, Fink B, Sandow D, Margull A, Berger I, Frommelt L (2008). Prolonged bacterial culture to identify late periprosthetic joint infection: a promising strategy. Clin Infect Dis.

[bib1.bib30] Shah NB, Tande AJ, Patel R, Berbari EF (2015). Anaerobic prosthetic joint infection. Anaerobe.

[bib1.bib31] She RC, Romney MG, Jang W, Walker T, Karichu JK, Richter SS (2018). Performance of the BacT/Alert Virtuo Microbial Detection System for the culture of sterile body fluids: prospective multicentre study. Clin Microbiol Infect.

[bib1.bib32] Suren C, Harrasser N, Pohlig F, Banke IJ, Lenze U, Lenze F, Knebel C, Von Eisenhart-Rothe R, Schauwecker J, Mühlhofer HML (2017). Prospective Analysis of a Sterile, Semi-automated Tissue Biopsy Homogenization Method in the Diagnosis of Prosthetic Joint Infections. In Vivo.

[bib1.bib33] Tande AJ, Patel R (2014). Prosthetic joint infection. Clin Microbiol Rev.

[bib1.bib34] Tarabichi M, Shohat N, Goswami K, Alvand A, Silibovsky R, Belden K, Parvizi J (2018). Diagnosis of Periprosthetic Joint Infection: The Potential of Next-Generation Sequencing. J Bone Joint Surg Am.

[bib1.bib35] Trampuz A, Zimmerli W (2006). Diagnosis and treatment of infections associated with fracture-fixation devices. Injury.

[bib1.bib36] Trampuz A, Gilomen A, Fluckiger U, Frei R, Zimmerli W, Widmer A (2005). Treatment outcome of infection associated with internal fixation devices: Results from a 5-year retrospective study (1999–2003).

[bib1.bib37] van den Bijllaardt W, van der Jagt OP, Peijs M, Janssens M, Buiting AG, Reuwer AQ (2019). Culturing periprosthetic tissue in blood culture bottles results in isolation of additional microorganisms. Eur J Clin Microbiol Infect Dis.

[bib1.bib38] Vasoo S (2018). Improving the Diagnosis of Orthopedic Implant-Associated Infections: Optimizing the Use of Tools Already in the Box. J Clin Microbiol.

[bib1.bib39] Velay A, Schramm F, Gaudias J, Jaulhac B, Riegel P (2010). Culture with BACTEC Peds Plus bottle compared with conventional media for the detection of bacteria in tissue samples from orthopedic surgery. Diagn Microbiol Infect Dis.

[bib1.bib40] Yano MH, Klautau GB, da Silva CB, Nigro S, Avanzi O, Mercadante MT, Salles MJ (2014). Improved diagnosis of infection associated with osteosynthesis by use of sonication of fracture fixation implants. J Clin Microbiol.

