# Supplementary material for: A prospective evaluation of the inoculation of homogenised tissue and bone biopsies in blood culture bottles for the diagnosis of orthopaedic-device-related infections
Source: J Bone Jt Infect. 2025 Aug 21;10(4):317–26. doi: 10.5194/jbji-10-317-2025 (PMC12590579; doi:10.5194/jbji-10-317-2025)
Supplement: The supplement related to this article is available online at https://doi.org/10.5194/jbji-10-317-2025-supplement. [file jbji-10-317-2025-supplement.pdf]

Supplement of J. Bone Joint Infect., 10, 317–326, 2025
https://doi.org/10.5194/jbji-10-317-2025-supplement
© Author(s) 2025. CC BY 4.0 License.

Supplement of

A prospective evaluation of the inoculation of homogenised tissue and bone biopsies in blood culture bottles for the diagnosis of orthopaedic-device-related infections

Ann-Sophie Jacob et al.

Correspondence to: Melissa Depypere (melissa.depypere@uzleuven.be)

The copyright of individual parts of the supplement might differ from the article licence.

Supplementary material

Table S1. EBJIS definition for PJI (Mcnally et al., 2021)

**EBJIS criteria for the diagnosis of clinically suspected periprosthetic joint infection**			
	**Infection unlikely (all findings negative)**	**Infection likely** (two positive findings)[a]	**Infection confirmed (any positive finding)**
**Clinical and blood workup**			
Clinical features	Clear alternative reason for implant dysfunction (e.g. fracture, implant breakage, malposition, tumour)	1. Radiological signs of loosening within the first 5 yrs after implantation<br>2. Previous wound healing problems<br>3. History of recent fever or bacteraemia<br>4. Purulence around the prosthesis[b]	Sinus tract with evidence of communication to the joint or visualization of the prosthesis
CRP		> 10 mg/l (1 mg/dl)[c]	
**Synovial fluid cytological analysis**[d]			
Leukocyte count (cells/µl)[c]	≤ 1,500	> 1,500	> 3,000
PMN%[c]	≤ 65%	> 65%	> 80%
**Synovial fluid biomarkers**			
Alpha-defensin[e]			Positive immunoassay or lateral-flow assay
**Microbiology**[f]			
Aspiration fluid		Positive culture	
Intraoperative (fluid and tissue)	All cultures negative	Single positive culture[g]	≥ 2 positive samples with the same microorganism
Sonication[h] (CFU/ml)	No growth	> 1 CFU/ml of any organism [g]	> 50 CFU/ml of any organism
**Histology**[c,i]			

HPF (400× magnification)	Negative	Presence of ≥ 5 neutrophils in a single HPF	Presence of ≥ 5 neutrophils in ≥ 5 HPF
			Presence of visible microorganisms
**Others**			
Nuclear imaging	Negative 3-phase Isotope Bone Scan[c]	Positive WBC scintigraphy[j]	

a. Infection is only likely if there is a positive clinical feature or raised serum CRP together with another positive test (synovial fluid, microbiology, histology, or nuclear imaging).

b. Except in adverse local tissue reaction (ALTR) and crystal arthropathy cases.

c. Should be interpreted with caution when other possible causes of inflammation are present: gout or other crystal arthropathy, metallosis, active inflammatory joint disease (e.g. rheumatoid arthritis), periprosthetic fracture, or the early postoperative period.

d. These values are valid for hip and knee periprosthetic joint infection. Parameters are only valid when clear fluid is obtained and no lavage has been performed. Volume for the analysis should be > 250 µl, ideally 1 ml, collected in an EDTA containing tube and analyzed in < 1 h, preferentially using automated techniques. For viscous samples, pretreatment with hyaluronidase improves the accuracy of optical or automated techniques. In case of bloody samples, the adjusted synovial WBC = synovial $WBC_{observed}$ – (WBC_{blood}/RBC_{blood} × $RBC_{synovial\ fluid}$) should be used.

e. Not valid in cases of ALTR, haematomas, or acute inflammatory arthritis or gout.

f. If antibiotic treatment has been given (not simple prophylaxis), the results of microbiological analysis may be compromised. In these cases, molecular techniques may have a place. Results of culture may be obtained from preoperative synovial aspiration, preoperative synovial biopsies, or (preferred) from intraoperative tissue samples.

g. Interpretation of single positive culture (or < 50 UFC/ml in sonication fluid) must be cautious and taken together with other evidence. If a preoperative aspiration identified the same microorganism, they should be considered as two positive confirmatory samples. Uncommon contaminants or virulent organisms (e.g. *Staphylococcus aureus* or Gram-negative rods) are more likely to represent infection than common contaminants (such as coagulase-negative staphylococci, micrococci, or *Cutibacterium acnes*).

h. If centrifugation is applied, then the suggested cut-off is 200 CFU/ml to confirm infection. If other variations to the protocol are used, the published cut-offs for each protocol must be applied.

i. Histological analysis may be from preoperative biopsy, intraoperative tissue samples with either paraffin or frozen section preparation.

j. WBC scintigraphy is regarded as positive if the uptake is increased at the 20-hour scan, compared to the earlier scans (especially when combined with complementary bone marrow scan).

CFU, colony-forming units; EBJIS, European Bone and Joint Infection Society; EDTA, ethylenediaminetetraacetic acid; HPF, high power field; PMN%, percentage of polymorphonuclear neutrophils; RBC, red blood cell; WBC, white blood cell count.

Table S2. FRI consensus definition

Confirmatory and suggestive criteria for the diagnosis of FRI (Metsemakers et al., 2018)

Confirmatory criteria	Suggestive criteria
Clinical signs<br>- Fistula<br>- Sinus<br>- Wound breakdown<br>- Purulent drainage or the presence of pus	Clinical signs<br>- Local/systemic (e.g. local redness, swelling, fever)<br>- New-onset joint effusion<br>- Persistent, increasing or new-onset wound drainage
Microbiology<br>- Phenotypically indistinguishable pathogens, identified by culture from at least 2 separate deep tissue/implant specimens	Laboratory signs<br>- Increased serum inflammatory markers (WBC, CRP)
Histopathology<br>- Presence of microorganisms in deep tissue specimens, confirmed by using specific staining techniques for bacteria and fungi<br>- Presence of > 5 PMNs/HPF in chronic/late-onset cases	- Radiological and/or nuclear imaging signs<br>- Microbiology pathogenic microorganisms identified from a single deep tissue/implant specimen

Table S3. All included samples, encompassing all identified microorganisms and the classification of positive or contaminated samples

		All detected microorganisms		Positivity		Contamination	
**Type of infection**	**Number of samples**	**BCB**	**CM**	**BCB**	**CM**	**BCB**	**CM**
PJI	6	*S. epidermidis*	*S. epidermidis, S. capitis*	Yes	Yes	No	Yes
FRI	6	*S. anginosus, F. nucleatum, S. aureus, D. pneumosintes, P. micra, A. schaallii, H. kunzii*	*S. aureus, S. anginosus, S. epidermidis, S. hominis*	Yes	No	No	Yes
FRI	6	*S. aureus*	*S. aureus*	Yes	Yes	No	No
PJI	6	*S. aureus*	*S. aureus*	Yes	Yes	No	No
PJI	7	none	none	No	No	No	No
FRI	6	*S. aureus*	*S. aureus*	Yes	Yes	No	No
FRI	6	none	none	No	No	No	No
FRI	6	*C. acnes*	*C. acnes, S. epidermidis, R. mucilaginosa*	Yes	Yes	No	Yes
PJI	5	*S. capitis*	none	Yes	No	No	No
FRI	7	none	*C. acnes, M. luteus*	No	No	No	Yes
FRI	8	*S. caprae*	*S. caprae*	Yes	Yes	No	No
PJI	6	none	*Bacillus* species, *S. warneri, S. epidermidis, S. hominis*	No	No	No	Yes
FRI	6	none	*S. epidermidis*	No	No	No	Yes
FRI	6	none	*S. warneri, S. epidermidis*	No	No	No	Yes
PJI	8	none	*S. warneri, S. epidermidis, S. pasteuri*	No	No	No	Yes
FRI	5	*S. aureus, M. morganii, E. cloacae complex, Providencia* species	*S. aureus, M. morganii, E. cloacae complex, Providencia* species	Yes	Yes	No	No

FRI	6	*Bacillus* species*, S. warneri*	*Bacillus* species, *C. acnes, M. luteus, S. epidermidis*	Yes	Yes	Yes	Yes
FRI	6	*S. aureus, S. hominis, S. epidermidis*	*S. aureus, S. hominis, S. warneri*	Yes	Yes	Yes	Yes
PJI	3	*E. coli*	*E. coli*	Yes	Yes	No	No
FRI	8	*S. epidermidis*	*S. warneri, S. mitis, Bacillus* species, *C. acnes., M. osloensis, S. epidermidis*	Yes	Yes	No	Yes
PJI	5	*E. coli*	none	Yes	No	No	No
FRI	5	*E. faecalis*	*E. faecalis*	Yes	Yes	No	No
FRI	5	none	none	No	No	No	No
PJI	7	*S. hominis*	*S. epidermidis*	No	No	Yes	Yes
FRI	5	*S. epidermidis*	*S. epidermidis, C. acnes*	Yes	Yes	No	Yes
FRI	5	*S. aureus*	*S. aureus, S. hominis*	Yes	Yes	No	Yes
PJI	4	*E. coli*	*E. coli, S. hominis, E. faecalis, C. amycolatum*	Yes	Yes	No	Yes
FRI	5	*C. amycolatum, S. mitis*	*S. aureus, C. amycolatum*	No	Yes	Yes	No
FRI	5	none	*S. epidermidis*	No	No	No	Yes
PJI	8	*S. epidermidis, S. hominis*	*S. epidermidis*	Yes	Yes	Yes	No
PJI	4	*C. acnes*	none	Yes	No	No	No
FRI	6	*S. hominis*	*S. hominis, C. acnes*	No	No	Yes	Yes
FRI	4	none	none	No	No	No	No
FRI	5	*S. epidermidis*	*S. epidermidis, S. hominis*	Yes	Yes	No	Yes
FRI	5	none	none	No	No	No	No
FRI	5	none	*C. acnes, Bacillus* species, *R. dentocariosa*	No	No	No	Yes
FRI	5	*S. epidermidis*	*S. epidermidis, S. capitis*	Yes	No	No	Yes
PJI	4	*P. aeruginosa*	*P. aeruginosa, S. warneri, A. fumigatus*	Yes	Yes	No	Yes

FRI	9	*P. aeruginosa*	*P. aeruginosa*	Yes	Yes	No	No
FRI	5	*Bacillus* species	*C. acnes, S. epidermidis*	No	Yes	Yes	Yes
FRI	5	*Streptococcus group C, E. cloacae complex, S. aureus*	*Streptococcus group C, E. cloacae complex, S. aureus*	Yes	Yes	No	No
FRI	5	*S. aureus*	*S. aureus*	Yes	Yes	No	No
PJI	6	*C. acnes*	*S. saprophyticus, M. luteus, C. acnes*	Yes	Yes	No	Yes
PJI	11	*S. mitis*	11	Yes	Yes	No	No
FRI	5	*P. aeruginosa, S. epidermidis*	*S. epidermidis*	Yes	No	No	No
FRI	5	*S. aureus*	*S. aureus*	Yes	Yes	No	No
FRI	5	none	*Bacillus species, S. warneri, S. pasteuri*	No	No	No	Yes
FRI	5	*S. epidermidis*	*S. hominis, M. luteus, C. acnes*	No	Yes	Yes	Yes
FRI	5	neg	none	No	No	No	No
FRI	5	*P. aeruginosa, K. oxytoca*	*P. aeruginosa, K. oxytoca*	Yes	Yes	No	No
PJI	9	*E. coli*	*E. coli*	Yes	Yes	No	No
FRI	5	none	*S. epidermidis*	No	Yes	No	No
FRI	3	*S. epidermidis*	*S. epidermidis*	Yes	Yes	No	No
FRI	5	none	*S. epidermidis, Bacillus* species	No	No	No	Yes
FRI	5	*C. metapsilosis, S. pettenkoferi*	*C. metapsilosis*	Yes	No	No	No
FRI	5	*S. epidermidis*	*P. stuartii, S. capitis*	No	No	Yes	Yes
FRI	5	*S. epidermidis*	*S. epidermidis, C. acnes, S. warneri*	No	Yes	No	Yes
FRI	5	none	*D. niter*	No	No	No	Yes
PJI	6	*S. agalactiae*	*S. agalactiae*	Yes	Yes	No	No

Abbreviations: *Staphylococcus epidermidis, Staphylococcus capitis, Streptococcus anginosus, Fusobacterium nucleatum, Staphylococcus aureus, Dialister pneumosintes, Parvimonas micra, Actinotignum schaallii, Helcococcus kunzii, Staphylococcus hominis, Cutibacterium acnes, Rothia mucilaginosa, Micrococcus luteus, Staphylococcus caprae, Staphylococcus warneri, Staphylococcus pasteuri, Morganella morganii, Enterobacter cloacae complex, Escherichia coli, Streptococcus mitis, Moraxella osloensis, Enterococcus faecalis, Corynebacterium amycolatum, Rothia dentocariosa, Pseudomonas aeruginosa, Aspergillus fumigatus,*

Staphylococcus saprophyticus, Klebsiella oxytoca, Candida metapsilosis, Staphylococcus pettenkoferi, Pseudomonas stuartii, Streptococcus agalactiae, Desulfovibrio niter.

Table S4. All positive cultures with at least one method

Positive culture blood culture bottles		Positive culture conventional method	
**Pathogenic microorganism**	**Contaminant**	**Pathogenic microorganism**	**Contaminant**
S. epidermidis		*S. epidermidis*	*S. capitis*
S. anginosus, F. nucleatum, S. aureus, D. pneumosintes, P. micra, A. schaallii, H. kunzii		*S. aureus, S. anginosus*	*S. epidermidis, S. hominis*
S. aureus		*S. aureus*	
S. aureus		*S. aureus*	
S. aureus		*S. aureus*	
C. acnes		*C. acnes*	*S. epidermidis, R. mucilaginosa*
S. capitis			
S. caprae		*S. caprae*	
S. aureus, M. morganii, E. cloacae complex, Providencia species		*S. aureus, M. morganii, E. cloacae complex, Providencia* species	
Bacillus species	*S. warneri*	*Bacillus* species	*C. acnes, M. luteus, S. epidermidis*
S. aureus	*S. hominis, S. epidermidis*	*S. aureus*	*S. warneri, S. hominis*
E. coli		*E. coli*	
S. epidermidis		*S. epidermidis*	*S. warneri, S. mitis, Bacillus* species, *C. acnes., M. osloensis*
E. coli			
E. faecalis		*E. faecalis*	
S. epidermidis		*S. epidermidis*	*C. acnes*
S. aureus		*S. aureus*	*S. hominis*
E. coli		*E. coli*	*S. hominis, E. faecalis, C. amycolatum*
	C. amycolatum, S. mitis	*S. aureus*	*C. amycolatum*
S. epidermidis	*S. hominis*	*S. epidermidis*	
C. acnes			

S. epidermidis		*S. epidermidis*	*S. hominis*
S. epidermidis		*S. epidermidis*	*S. capitis*
P. aeruginosa		*P. aeruginosa*	*A. fumigatus, S. warneri*
P. aeruginosa		*P. aeruginosa*	
	Bacillus species	*S. epidermidis, C. acnes*	
Streptococcus group C, E. cloacae complex,S. aureus		*Streptococcus group C, E. cloacae complex, S. aureus*	
S. aureus		*S. aureus*	
C. acnes		*C. acnes*	*S. saprophyticus, M. luteus*
S. mitis		*S. mitis*	
P. aeruginosa, S. epidermidis		*S. epidermidis*	
S. aureus		*S. aureus*	
	S. epidermidis	*C. acnes*	*S. hominis, M. luteus*
P. aeruginosa, K. oxytoca		*P. aeruginosa, K. oxytoca*	
E. coli		*E. coli*	
		S. epidermidis	
S. epidermidis		*S. epidermidis*	
C. metapsilosis, S. pettenkoferi		*C. metapsilosis*	
S. epidermidis		*S. epidermidis, C. acnes*	*S. warneri*
S. agalactiae		*S. agalactiae*	
